# MiR-16-5p targets *SESN1* to regulate the *p53* signaling pathway, affecting myoblast proliferation and apoptosis, and is involved in myoblast differentiation

**DOI:** 10.1038/s41419-018-0403-6

**Published:** 2018-03-06

**Authors:** Bolin Cai, Manting Ma, Biao Chen, Zhenhui Li, Bahareldin Ali Abdalla, Qinghua Nie, Xiquan Zhang

**Affiliations:** 1 Department of Animal Genetics, Breeding and Reproduction, College of Animal Science, South China Agricultural University, Guangzhou, 510642 Guangdong China; 2 Guangdong Provincial Key Lab of Agro-Animal Genomics and Molecular Breeding, and Key Laboratory of Chicken Genetics, Breeding and Reproduction, Ministry of Agriculture, Guangzhou, 510642 Guangdong China; 3 National-Local Joint Engineering Research Center for Livestock Breeding, Guangzhou, 510642 Guangdong China

## Abstract

The proliferation, apoptosis, and differentiation of myoblasts are essential processes in skeletal muscle development. During this developmental process, microRNAs (miRNAs) play crucial roles. In our previous RNA-seq study (accession number GSE62971), we found that miR-16-5p was differentially expressed between fast and slow growth in chicken. In this study, we report that miR-16-5p could inhibit myoblast proliferation, promote myoblast apoptosis, and repress myoblast differentiation by directly binding to the 3′ UTR of *SESN1*, which is also differentially expressed. Overexpression of *SESN1* significantly promoted the proliferation, inhibited apoptosis, and induced differentiation of myoblasts. Conversely, its loss of function hampered myoblast proliferation, facilitated myoblast apoptosis, and inhibited myoblast differentiation. Interestingly, we found *SESN1* could regulate *p53* by a feedback mechanism, thereby participating in the regulation of *p53* signaling pathway, which suggests that this feedback is indispensable for myoblast proliferation and apoptosis. Altogether, these data demonstrated that miR-16-5p directly targets *SESN1* to regulate the *p53* signaling pathway, and therefore affecting myoblast proliferation and apoptosis. Additionally, *SESN1* targets myogenic genes to control myoblast differentiation.

## Introduction

Since the first microRNA (miRNA) Lin-4 was discovered in nematode in 1993^1^, and the function of miRNA let-7 was subsequently demonstrated^2^, miRNAs began to attract the attention of researchers and subsequently they have become an intense and focused area of biological research. MicroRNAs are endogenous noncoding single-stranded RNA molecules of 21 to 23 nucleotides long^3^ that are capable of degrading or inhibiting target mRNAs by perfect or imperfect pairing with the 3′ untranslated region (3′ UTR) of the target mRNA to regulate post-transcriptional gene expression^4^. In animal models, 740 miRNA precursors have been mapped to the chicken genome in miRBase 21, which result in 994 mature miRNAs.

Muscle formation is a process in which myoblasts withdraw from the cell cycle, express muscle-specific genes, and prevent the expression of other cell- or tissue-specific genes. Many miRNAs have been found to regulate muscle developmental processes in several different ways^5–7^. The first evidence that miRNAs were involved in muscle development came from the accumulation of specific miRNAs in muscle cells^8^. A recent study analyzed chicken miRNAs by computer prediction and found 33 new and 189 known miRNAs. During this analysis, 17 differentially expressed miRNAs were found in broilers and laying hens that may be associated with muscle development. Through miRNA target prediction and network analysis, it was found that these miRNAs could affect muscle growth of broilers and layers by targeting *Activin A Receptor Type 2B* (*ACVR2B*)^9^. During the same developmental stage, miR-1623, miR-181b, let-7b, and miR-128 were differentially expressed in the skeletal muscle of dwarf and normal chickens^10^.

Gga-miR-16-5p is the mature miRNA which results from the two precursor miRNAs (gga-miR-16-1 and gga-miR-16-2), with a mature sequence of 22 nt. Recent studies have shown that miR-16-5p plays a regulatory role in the molecular machinery that enhances muscle protein synthesis in response to protein ingestion following concurrent exercise^11^. However, how Gga-miR-16-5p regulates the development of skeletal muscle in chicken is still unknown.

It is well established that the tumor suppressor gene *p53* is a key component in the induction of cell cycle arrest and apoptosis^12^. The p53 protein is an important transcription factor that regulates growth arrest, apoptosis, and DNA repair in response to various stress stimuli^13^. *SESN1* (Sestrin 1) is located on the third chromosome of the chicken genome, and is involved in the *p53* signaling pathway. SESN1 is a member of a highly conserved family of stress-induced proteins. In mammals, this family is composed of three members: SESN1, SESN2, and SESN3. Compared to the various tissues of adult animals, *SESN1* and *SESN2* had the highest expression in skeletal muscle^14^. An *SESN1* mutant *Caenorhabditis elegans* demonstrated severely damaged muscle cells as indicated by abnormal orientation of muscle actin fibers^15^. Moreover, loss of *Drosophila* sestrin could result in degeneration of thoracic muscles with loss of sarcomeric structure, including discontinued Z discs, disappearance of M bands, scrambled actomyosin arrays, and diffused sarcomere boundaries^14^.

White recessive rock (WRR) is a broiler chicken with a fast growth rate, which exhibits a different growth performance from Xinghua (XH) chicken (a Chinese native breed with a slow growth rate) at 7 weeks of age^16^. In our previous RNA-seq study, we found that both miR-16-5p (accession number GSE62971)^16^ and *SENS1* (accession number GSE72424)^17^ were differentially expressed between WRR and XH chickens (Supplementary File 1). To study the role of miR-16-5p in chicken skeletal muscle development, we explored its molecular function and found that miR-16-5p could directly suppress *SESN1* to regulate myoblast proliferation and apoptosis via the *p53* signaling pathway. Additionally, we also confirmed that miR-16-5p was involved in myoblast differentiation by targeting *SESN1*.

## Results

### miR-16-5p represses myoblast proliferation

**Fig. 1:**
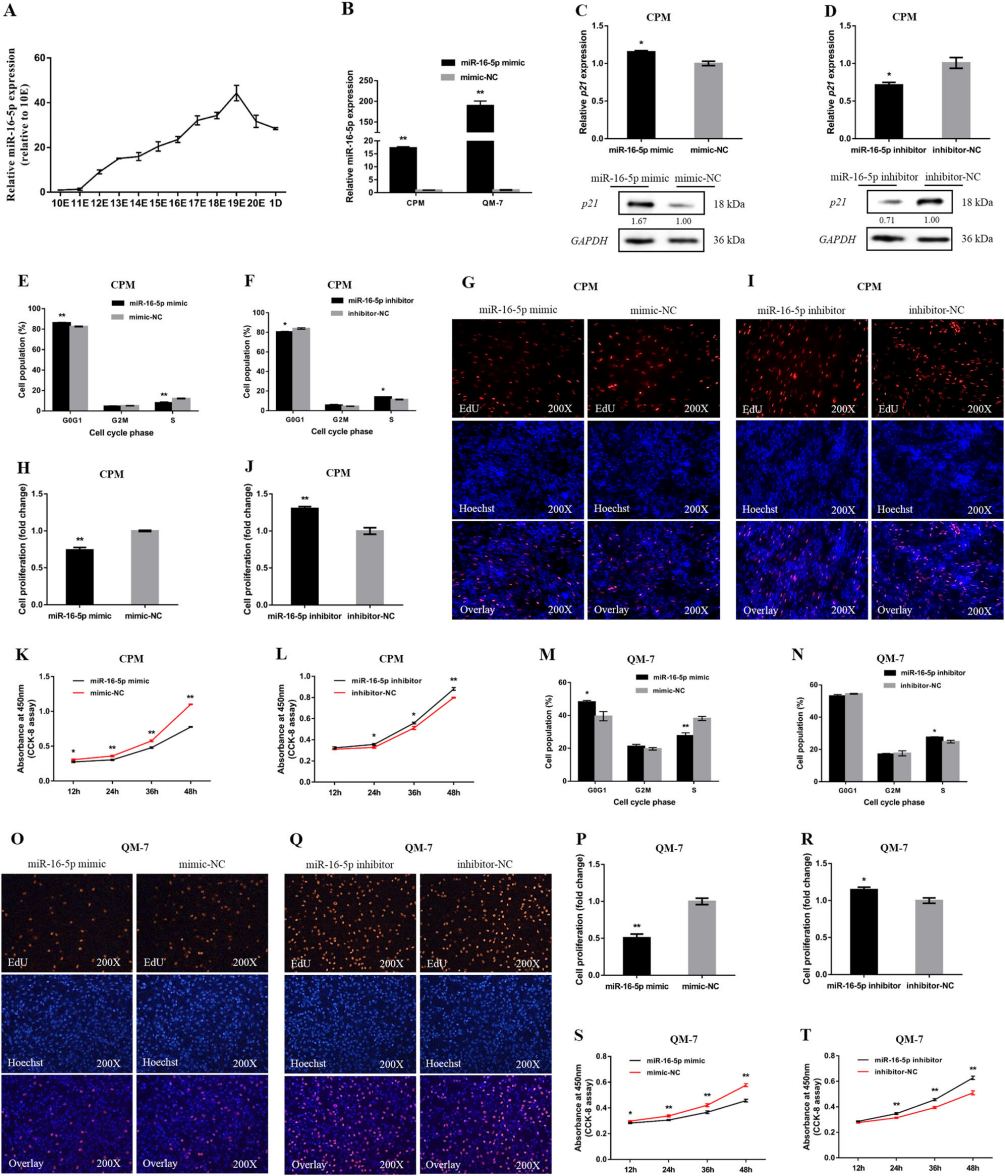
miR-16-5p inhibits myoblast proliferation. **a** The relative expression of miR-16-5p in chicken embryonic breast muscle. **b** The relative expression of miR-16-5p from miR-16-5p mimic transfected CPMs and QM-7 cells. **c**, **d** The relative mRNA and protein expression of *p21* after transfection with miR-16-5p mimic and inhibitor in chicken CPMs. The numbers shown below bands were folds of band intensities relative to control. Band intensities were quantified by ImageJ and normalized to *GAPDH*. Data are expressed as a fold change relative to the control. **e**,** f** Cell cycle analysis of CPMs 48 h after overexpression and inhibition of miR-16-5p, using propidium iodide staining for DNA content. **g**, **i** Proliferation of transfected CPMs was assessed by EdU incorporation. **h**, **j** Proliferation rates of CPMs with miR-16-5p overexpression and inhibition. **k**, **l** Cell growth was measured following the transfection of miR-16-5p mimic and inhibitor in CPMs. **m**, **n** Cell cycle analysis of QM-7 cells 48 h after overexpression and inhibition of miR-16-5p. **o**, **q** Proliferation of transfected QM-7 cells was assessed by EdU incorporation. **p**,** r** Proliferation rates of QM-7 cells with miR-16-5p overexpression and inhibition. **s**, **t** Cell growth was measured following the transfection of miR-16-5p mimic and inhibitor in QM-7 cells. In all panels, the results are shown as mean ± S.E.M. and the data are representative of three independent assays. Statistical significance of differences between means was assessed using an unpaired Student’s *t*-test (*P < 0.05; **P < 0.01) vs. NC, negative control

During breast muscle development in XH chickens, miR-16-5p was expressed at embryonic days 11 (11E) and subsequently increased and peaked at 19E (Fig. 1a). The increased expression of miR-16-5p in embryonic development suggested that miR-16-5p was probably involved in skeletal muscle development.

To unveil the functions of miR-16-5p, we performed overexpression and inhibition experiments to assess its role in cell proliferation and viability. The relative expressions of miR-16-5p were detected after 48 h of transfection with miR-16-5p mimic (Fig. 1b). In chicken primary myoblast (CPM), overexpression of miR-16-5p promoted *p21* mRNA and protein expression, and significantly increased the number of cells that progressed to G0/G1 and reduced the number of S phase cells (Fig. 1c, e). Conversely, miR-16-5p inhibition significantly downregulated the expression of *p21* and resulted in a fewer number of G0/G1 and increased S phase cells (Fig. 1d, f). In addition, the 5-ethynyl-2′-deoxyuridine (EdU) assay and cell counting kit-8 (CCK-8) assay demonstrated that miR-16-5p overexpression significantly repressed myoblast viability, while its inhibition promoted their proliferation (Fig 1g–l).

We found similar results in quail muscle clone 7 (QM-7). MiR-16-5p overexpression significantly increased the number of cells in G0/G1, and significantly decreased the number of S phase cells (Fig. 1m). This also resulted in a significant repress in myoblast proliferation, as judged by EdU incorporation and CCK-8 assay (Fig. 1o, p, s). On the contrary, G0/G1 cells decreased while S phase cells increased significantly (Fig. 1n), and proliferation was significantly promoted after miR-16-5p inhibition (Fig. 1q, r, t).

### miR-16-5p promotes myoblast apoptosis

**Fig. 2:**
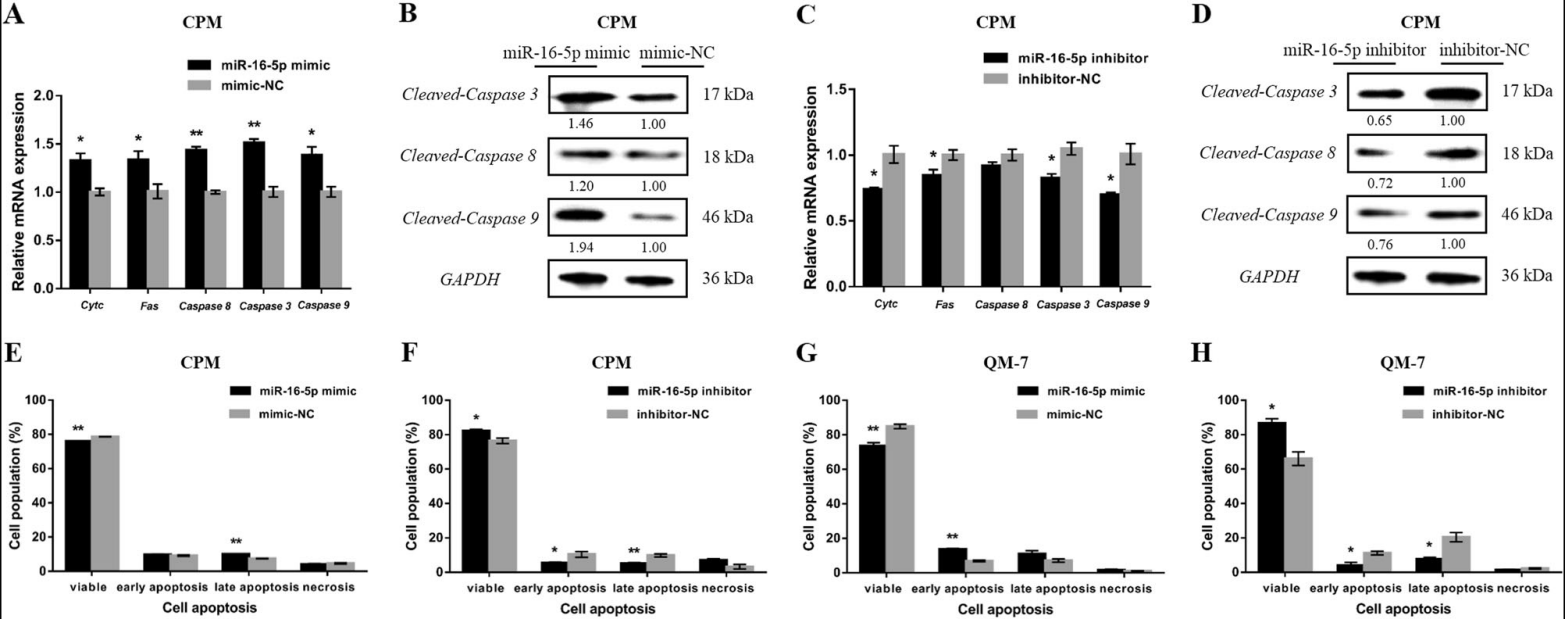
miR-16-5p facilitates myoblast apoptosis. **a**,** c** mRNA levels of several apoptosis-related genes induced by miR-16-5p overexpression and inhibition in CPMs. **b**,** d** The protein expression levels of several cleaved caspases with miR-16-5p overexpression and inhibition in CPMs. The numbers shown below bands were folds of band intensities relative to control. Band intensities were quantified by ImageJ and normalized to *GAPDH*. Data are expressed as a fold change relative to the control. **e**,** f** Annexin V-FITC and propidium iodide (PI) dual staining detection of the apoptosis of CPMs after overexpression and inhibition of miR-16-5p, as determined by flow cytometry. **g**,** h** Annexin V-FITC and PI dual staining detection of the apoptosis of QM-7 cells after overexpression and inhibition of miR-16-5p, as determined by flow cytometry. In all panels, data are presented as mean ± S.E.M. of three biological replicates. Statistical significance of differences between means was assessed using an unpaired Student’s *t*-test (*P < 0.05; **P < 0.01) vs. NC, negative control

To verify the biological effects of miR-16-5p on myoblast apoptosis, the mRNA expression levels of several apoptosis-related genes, including *Cytochrome c* (*Cyt c*), *Fas*, *Caspase 8*, *Caspase 3*, and *Caspase 9*, were examined by quantitative PCR (qPCR) after overexpression or inhibition of miR-16-5p in CPM. Furthermore, the protein levels of Cleaved-Caspase 3, Cleaved-Caspase 8, and Cleaved-Caspase 9 were also detected by western blot. The results showed that overexpression of miR-16-5p could promote the upregulation and activation of apoptosis-related genes (Fig. 2a, b). In contrast, apoptosis-related genes were downregulated and inactivated after inhibition of miR-16-5p (Fig. 2c, d).

In addition, myoblast apoptosis was analyzed by flow cytometry using a conjugated Annexin V antibody and propidium iodide (PI) staining. In both CPM and QM-7 cells, overexpression of miR-16-5p promoted myoblast apoptosis, as revealed by a higher apoptotic cell ratio and fewer viable cells (Fig. 2e, g). Conversely, miR-16-5p inhibition repressed myoblast apoptosis, suggesting that miR-16-5p has a positive regulatory effect on myoblast apoptosis (Fig 2f, h).

### miR-16-5p represses myoblast differentiation

**Fig. 3:**
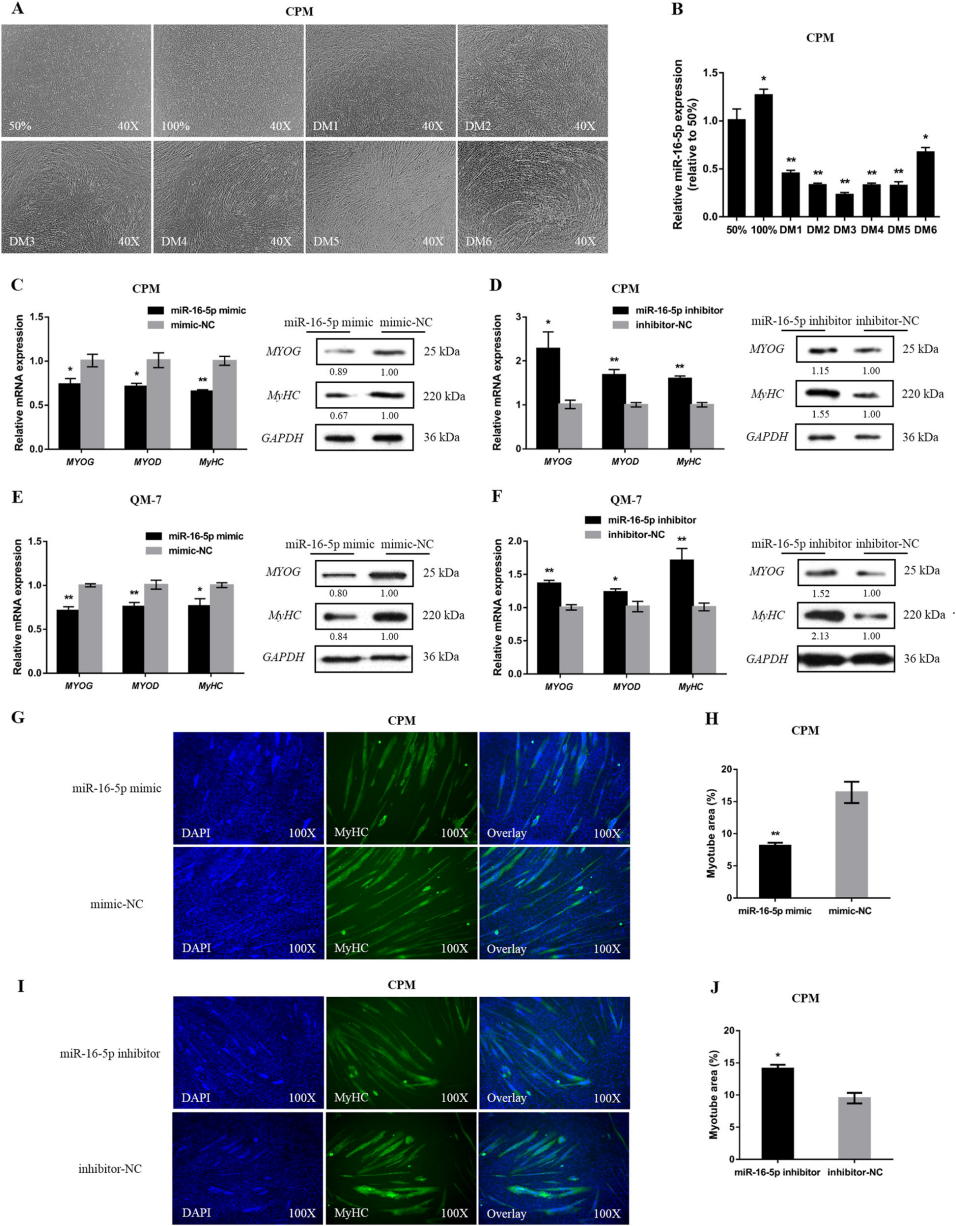
miR-16-5p inhibits myoblast differentiation. **a** Microscopic images of CPMs during proliferation (50% and 100% confluency) and differentiation (CPMs cultured in differentiation medium from 1 to 6 day [DM1 to DM6; DM indicate differentiation day]) periods. **b** The relative expression of miR-16-5p during CPM differentiation. **c**, **d** The mRNA and protein expression levels of myoblast differentiation marker genes from miR-16-5p mimic and inhibitor transfected CPMs. The numbers shown below bands were folds of band intensities relative to control. Band intensities were quantified by ImageJ and normalized to *GAPDH*. Data are expressed as a fold change relative to the control. **e**, **f** The mRNA and protein expression levels of myoblast differentiation marker genes with miR-16-5p overexpression and inhibition in QM-7 cells. The numbers shown below bands were folds of band intensities relative to control. Band intensities were quantified by ImageJ and normalized to *GAPDH*. Data are expressed as a fold change relative to the control. **g**, **i** *MyHC* staining of myoblasts at 72 h after transfection of miR-16-5p mimic and inhibitor in CPMs. **h**, **j** Myotube area (%) of CPMs 72 h after overexpression and inhibition of miR-16-5p. In all panels, data are presented as means ± S.E.M. of three independent experiments, and statistical significance of differences between means was assessed using an unpaired Student’s *t*-test (*P < 0.05; **P < 0.01) vs. NC, negative control

To further investigate the potential roles of miR-16-5p, CPMs were induced to differentiate in vitro (Fig. 3a). As differentiation progressed, the expression level of miR-16-5p significantly decreased, which suggested that miR-16-5p was involved in the process of myoblast differentiation (Fig. 3b).

Meanwhile, myoblast differentiation marker genes, including *MYOG*, *MYOD*, and *MyHC*, were analyzed by qPCR after overexpression or inhibition of miR-16-5p in both CPM and QM-7 cells. And the protein levels of *MYOG* and *MyHC* were also examined by western blot. The expressions of these marker genes were all significantly downregulated in the miR-16-5p mimic transfected cells compared to control cells (Fig. 3c, e). Conversely, inhibition of miR-16-5p promoted their expression (Fig. 3d, f). Moreover, we transfected CPMs with miR-16-5p mimic or inhibitor, and then induced myoblast differentiation. After immunofluorescence staining, we found miR-16-5p overexpression repressed myoblast differentiation and significantly reduced the total areas of myotubes (Fig. 3g, h), while inhibition of miR-16-5p promoted myoblast differentiation (Fig. 3I, j).

### SESN1 is a direct target of miR-16-5p

**Fig. 4:**
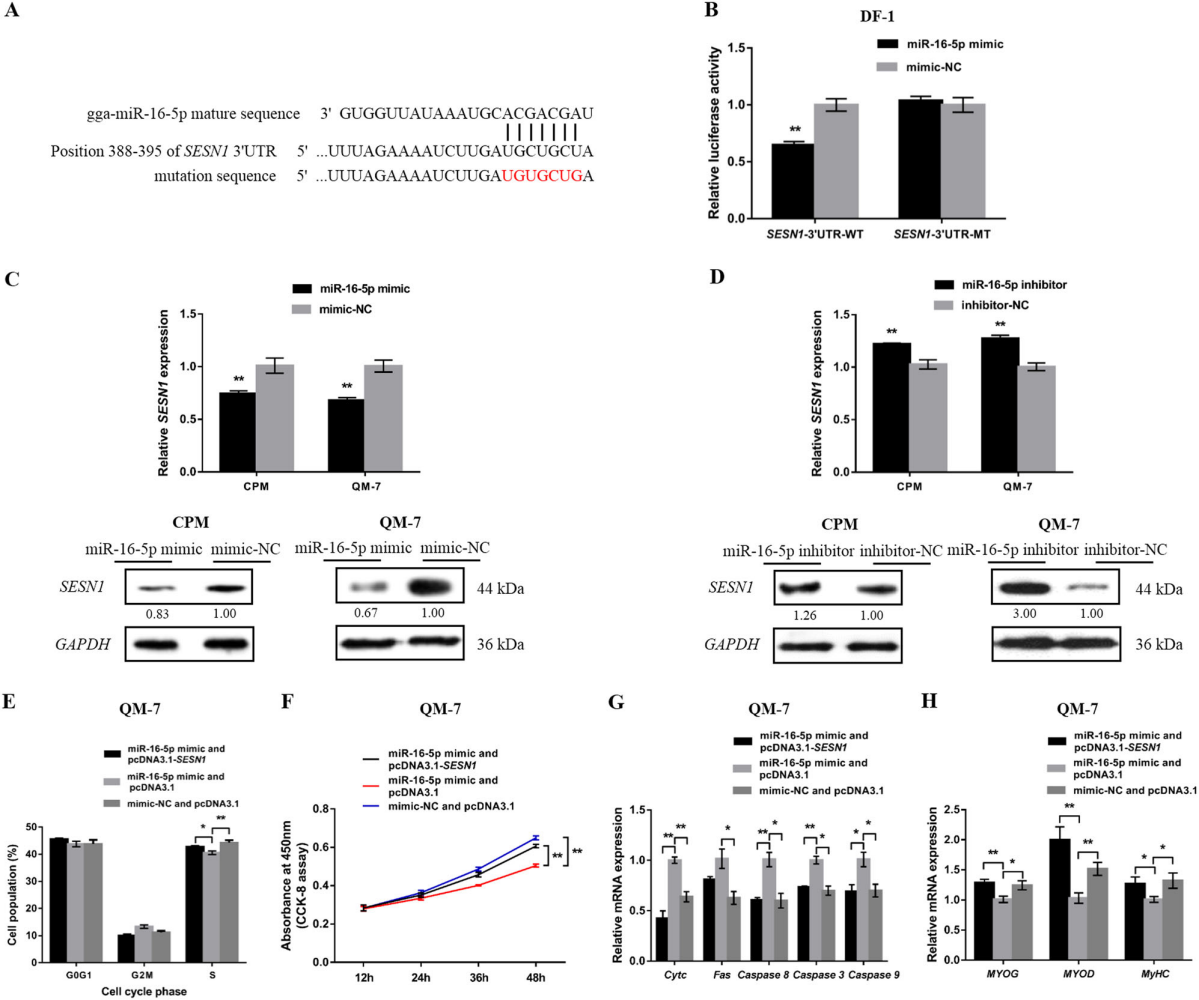
Identification of *SESN1* as a direct target of miR-16-5p. **a** The potential binding site of miR-16-5p in the *SESN1* mRNA 3′ UTR. The mutant sequence in miR-16-5p binding site is highlighted in red. **b** Luciferase assay was conducted by co-transfecting wild type or mutant *SESN1* 3′ UTR with miR-16-5p mimic or mimic-NC in DF-1 cells. **c**,** d** The mRNA and protein expression levels of *SESN1* from miR-16-5p mimic and inhibitor transfected CPMs and QM-7 cells. The numbers shown below bands were folds of band intensities relative to control. Band intensities were quantified by ImageJ and normalized to *GAPDH*. Data are expressed as a fold change relative to the control. **e** Cell cycle analysis of QM-7 cells after co-transfection with the listed nucleic acids. **f** QM-7 cells growth curves following transfection of the listed nucleic acids. **g** mRNA levels of several apoptosis-related genes after co-transfection with the listed nucleic acids in QM-7 cells. **h** The mRNA expression levels of myoblast differentiation marker genes from co-transfected QM-7 cells. In all panels, results are expressed as the mean ± S.E.M. of three independent experiments, and statistical significance of differences between means was assessed using an unpaired Student’s *t*-test (*P < 0.05; **P < 0.01) vs. NC, negative control

In order to further understand the molecular mechanism by which miR-16-5p regulates gene expression, we predicted its target genes on the miRDB (http://www.mirdb.org/miRDB/) and TargetScanHuman 7.1 (http://www.targetscan.org/vert_71/) databases. The results showed that the seed sequence of miR-16-5p could perfectly match the 3′ UTR position 388–395 of chicken *SESN1* mRNA, which suggested *SESN1* was a potential target of miR-16-5p (Fig. 4a). To confirm whether miR-16-5p directly targets the 3′ UTR of *SESN1*, a dual-luciferase reporter assay was carried out in embryonic chicken fibroblast cell line DF-1 cells. The recombinant reporter vectors (pmirGLO-*SESN1*-WT and pmirGLO-*SESN1*-MT) were co-transfected with miR-16-5p mimic or mimic-normal control (NC). We found that the luciferase activity of the wild-type group (*SESN1*-3′ UTR-WT) was significantly decreased after transfection with miR-16-5p mimic, whereas no significant difference was observed in the mutant group (*SESN1*-3′ UTR-MT) (Fig. 4b). More importantly, after overexpression of miR-16-5p, the mRNA and protein levels of *SESN1* were significantly decreased, while inhibition of miR-16-5p upregulated expression of *SESN1* mRNA and protein (Fig. 4c, d). This was suggestive that miR-16-5p was a potential regulatory factor of *SESN1*.

To further evaluate the roles of *SESN1* in the functional effects of miR-16-5p, we co-transfected myoblasts with; (a) miR-16-5p mimic and pcDNA3.1-*SESN1*, (b) miR-16-5p mimic and pcDNA3.1, and (c) mimic-NC and pcDNA3.1-*SESN1* to study their effects on myoblast proliferation, apoptosis, and differentiation. The results showed that overexpression of miR-16-5p inhibited the proliferation of myoblasts, promoted the expression of apoptosis-related genes, and downregulated myoblast differentiation marker genes. However, these regulatory roles were negated by co-overexpression of miR-16-5p and *SESN1* (Fig. 4e–h). Taken together, these results demonstrated that *SESN1* was a direct target of miR-16-5p.

### SESN1 promotes proliferation and represses apoptosis of myoblast via the p53 signaling pathway

**Fig. 5:**
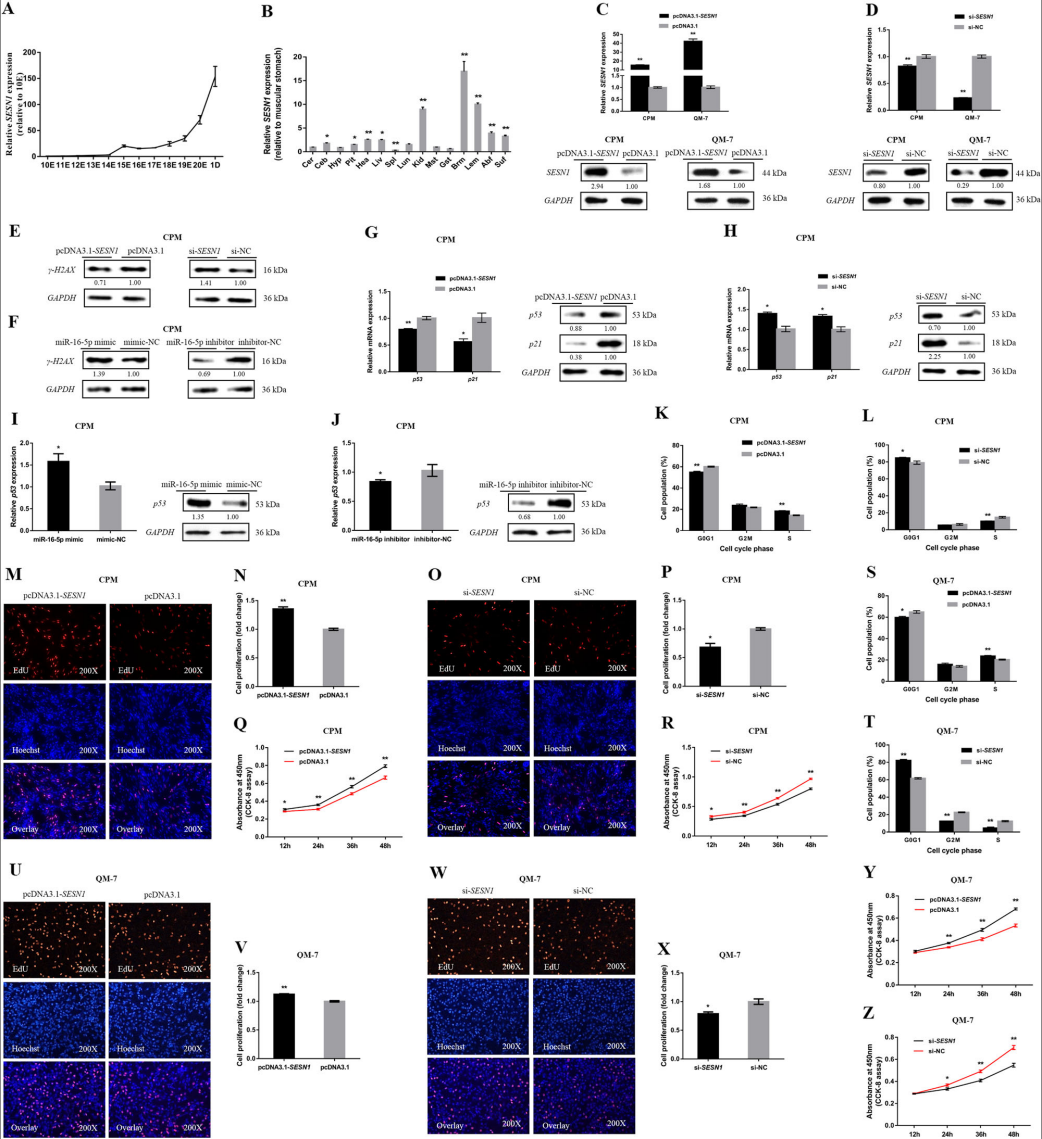
*SESN1* facilitates myoblast proliferation via the *p53* signaling pathway. **a** The relative mRNA expression of *SESN1* in chicken embryonic breast muscle. **b** Xinghua (XH) chicken tissue expression profiles of *SESN1*. The horizontal axis and vertical axis indicate different tissues and their relative expression values, respectively (mean ± S.E.M). Cer cerebrum, Ceb cerebellum, Hyp hypothalamus, Pit pituitary, Hea heart, Liv liver, Spl spleen, Lun lung, Kid kidney, Mst muscular stomach, Gst glandular stomach, Brm breast muscle, Lem leg muscle, Abf abdominal fat, and Suf subcutaneous fat. **c**,** d** The mRNA and protein expression levels of *SESN1* with *SESN1* overexpression and knockdown in CPMs and QM-7 cells. The numbers shown below bands were folds of band intensities relative to control. Band intensities were quantified by ImageJ and normalized to *GAPDH*. Data are expressed as a fold change relative to the control. **e**, **f** Western blotting analysis of γ-H2AX protein levels after *SESN1* (**e**) and miR-16-5p (**f**) overexpression and knockdown in CPMs. The numbers shown below bands were folds of band intensities relative to control. Band intensities were quantified by ImageJ and normalized to *GAPDH*. Data are expressed as a fold change relative to the control. **g**,** h** The mRNA and protein expression levels of *p53* and *p21* after transfection of pcDNA3.1-*SESN1* and si-*SESN1* in CPMs. The numbers shown below bands were folds of band intensities relative to control. Band intensities were quantified by ImageJ and normalized to *GAPDH*. Data are expressed as a fold change relative to the control. **i**, **j** The mRNA and protein expression levels of *p53* after overexpression and inhibition of miR-16-5p in CPMs. The numbers shown below bands were folds of band intensities relative to control. Band intensities were quantified by ImageJ and normalized to *GAPDH*. Data are expressed as a fold change relative to the control. **k**,** l** Cell cycle analysis of CPMs after *SESN1* overexpression and knockdown. **m**,** o** EdU proliferation assays for CPMs with overexpression and inhibition of *SESN1.* **n**,** p** The numbers of proliferative cells were also counted. **q**, **r** CPMs growth curves following transfection of pcDNA3.1-*SESN1* and si-*SESN1*. **s**,** t** QM-7 cells were collected for cell cycle analysis 48 h after transfection. **u**,** w** EdU proliferation assays for QM-7 cells with overexpression and inhibition of *SESN1*, **v**,** x** the numbers of proliferative cells were also counted. **y**,** z** QM-7 cells growth curves following transfection of pcDNA3.1-*SESN1* and si-*SESN1*. In all panels, results are expressed as mean ± S.E.M. of three replicates. Statistical significance of differences between means was assessed using an unpaired Student’s *t*-test (*P < 0.05; **P < 0.01) vs. NC, negative control

During chicken breast muscle development, the expression of *SESN1* showed a general upward trend, and began to rise sharply by 19E (Fig. 5a). We also examined tissue expression profiles of *SESN1* and found high expression in breast and leg muscles (Fig. 5b). *SESN1* overexpression and knockdown experiments in CPMs and QM-7 cells were also performed to verify the regulatory functions of *SESN1* in myoblast (Fig. 5c, d). *SESN1*, as a DNA damage-inducible protein, was found to play a critical role in the repair of damaged DNA^18,19^. The phosphorylation of H2AX at ser139, or γ-H2AX, which is a marker of double-strand breaks (DSBs)^20–22^, was detected after *SESN1* overexpression or interference. Overexpression of *SESN1* facilitated DNA repair (Fig. 5e). Conversely, DNA damage was induced with the knockdown of *SESN1* (Fig. 5e). The contrary results were found with miR-16-5p overexpression or inhibition (Fig. 5f). In the meantime, *SESN1* is a member of the *p53* signaling pathway, which is also well-known to response to DNA damage and facilitate DNA repair^23–25^. So we also examined the expression of *p53* and *p21* after transfection of the pcDNA3.1-*SESN1* plasmid and si-*SESN1* fragments. Overexpression of *SESN1* significantly downregulated the expression of *p53*, whereas its loss of function increased *p53* expression (Fig. 5g, h). Similar results were found for *p21* expression (Fig. 5g, h). Moreover, we examined the expression of *p53* after overexpression or inhibition of miR-16-5p, and opposite results were observed compared to *SESN1* (Fig. 5i, j). Changes in *p53* expression suggested that *SESN1* may affect the repair of damaged DNA, thereby regulating the physiological function of myoblast via the *p53* signaling pathway.

In CPM, *SESN1* overexpression resulted in a decline in G0/G1 phase cells and an increased number of cells that progressed to S phase (Fig. 5k). In the meantime, myoblast proliferation was significantly promoted (Fig. 5m, n, q). However, the knockdown of *SESN1* resulted in a large number of G0/G1 and few S phase cells (Fig. 5l), and significant inhibition of myoblast viability (Fig. 5o, p, r). In QM-7 cells, *SESN1* overexpression also promoted myoblast proliferation and division (Fig. 5s, u, v, y). Conversely, its loss of function increased cell cycle arrest in the G0/G1 stage and inhibited the proliferation of myoblast, which was similar to what was observed in CPM (Fig. 5t, w, x, z).

**Fig. 6:**
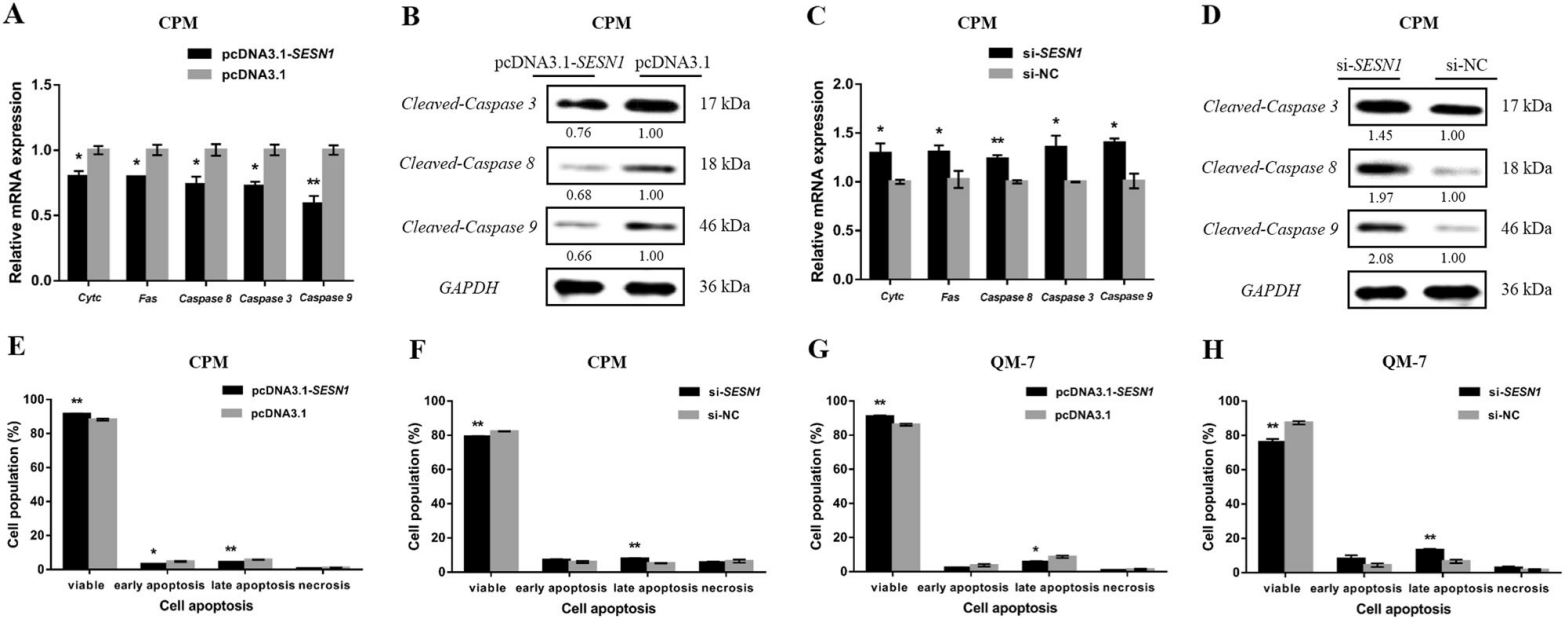
*SESN1* inhibits myoblast apoptosis *via* the *p53* signaling pathway. **a**,** c** The mRNA expression levels of several apoptosis-related genes with *SESN1* overexpression and knockdown in CPMs. **b**, **d** Western blotting analysis of several cleaved caspases after *SESN1* overexpression and inhibition in CPMs. The numbers shown below bands were folds of band intensities relative to control. Band intensities were quantified by ImageJ and normalized to *GAPDH*. Data are expressed as a fold change relative to the control. **e**–**h** Both CPMs and QM-7 cells were collected for the detection of apoptotic cells by flow cytometry, 48 h after transfection. In all panels, data are presented as means ± S.E.M. of three independent assays. Statistical significance of differences between means was assessed using an unpaired Student’s *t*-test (*P < 0.05; **P < 0.01) vs. NC, negative control

The *p53* signaling pathway is also involved in cell apoptosis. Therefore, we analyzed apoptosis-related gene expression after *SESN1* overexpression or knockdown. At the same time, the protein levels of Cleaved-Caspase 3, Cleaved-Caspase 8, and Cleaved-Caspase 9 were also elevated. Overexpression of *SESN1* suppressed the expression of apoptosis-related genes examined (Fig. 6a, b). Conversely, their expressions were significantly upregulated with the knockdown of *SESN1* (Fig. 6c, d). Additionally, the results of Annexin V-FITC and PI dual staining also indicated that *SESN1* could repress myoblast apoptosis (Fig. 6e–h).

### SESN1 is involved in myoblast differentiation

**Fig. 7:**
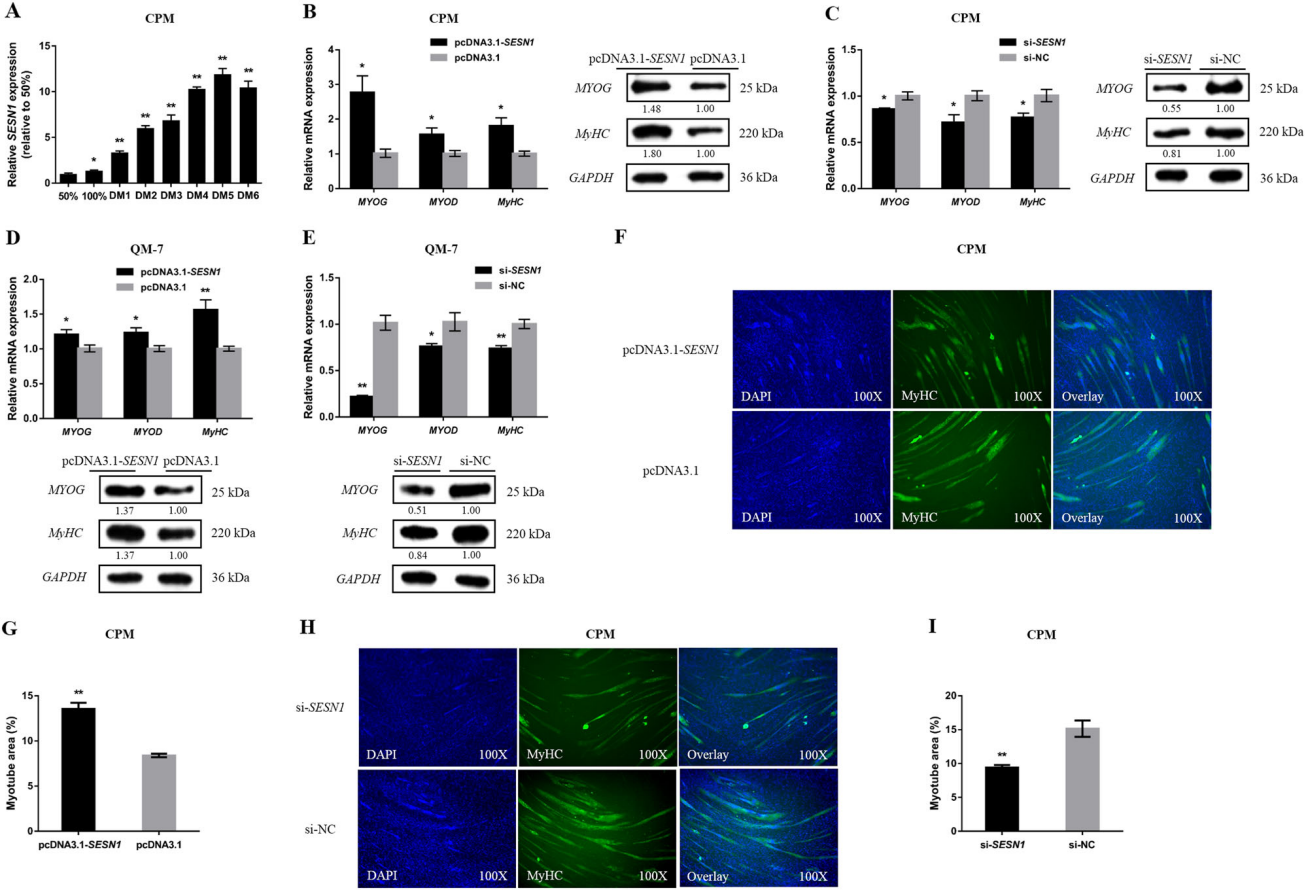
*SESN1* promotes myoblast differentiation. **a** The relative mRNA expression level of *SESN1* during CPM differentiation. **b**–**e** Both in CPMs and QM-7 cells, the mRNA and protein expression levels of myoblast differentiation marker genes with transfection of pcDNA3.1-*SESN1* and si-*SESN1* were detected. The numbers shown below bands were folds of band intensities relative to control. Band intensities were quantified by ImageJ and normalized to *GAPDH*. Data are expressed as a fold change relative to the control. **f**,** h** Immunofluorescence analysis of MyHC-staining cells after pcDNA3.1-*SESN1* (**f**) and si-*SESN1* (**h**) transfection into CPMs. **g**, **i** Myotube area (%) of CPMs with *SESN1* overexpression and knockdown. In all panels, results are expressed as the mean ± S.E.M. of three biological replicates. Statistical significance of differences between means was assessed using an unpaired Student’s *t*-test (*P < 0.05; **P < 0.01) vs. NC, negative control

After induction of CPM differentiation in vitro, the levels of *SESN1* expression showed a gradual decrease (Fig. 7a). Additionally, both in CPMs and in QM-7 cells, overexpression or knockdown of *SESN1* promoted or inhibited, respectively, myoblast differentiation marker-gene expression (Fig. 7b, e). Immunofluorescence staining showed that *SESN1* overexpression promoted myoblast differentiation in associated with increased total areas of myotubes (Fig. 7f, g). On the contrary, myoblast differentiation was significantly repressed after *SESN1* inhibition (Fig. 7h, i).

## Discussion

**Fig. 8:**
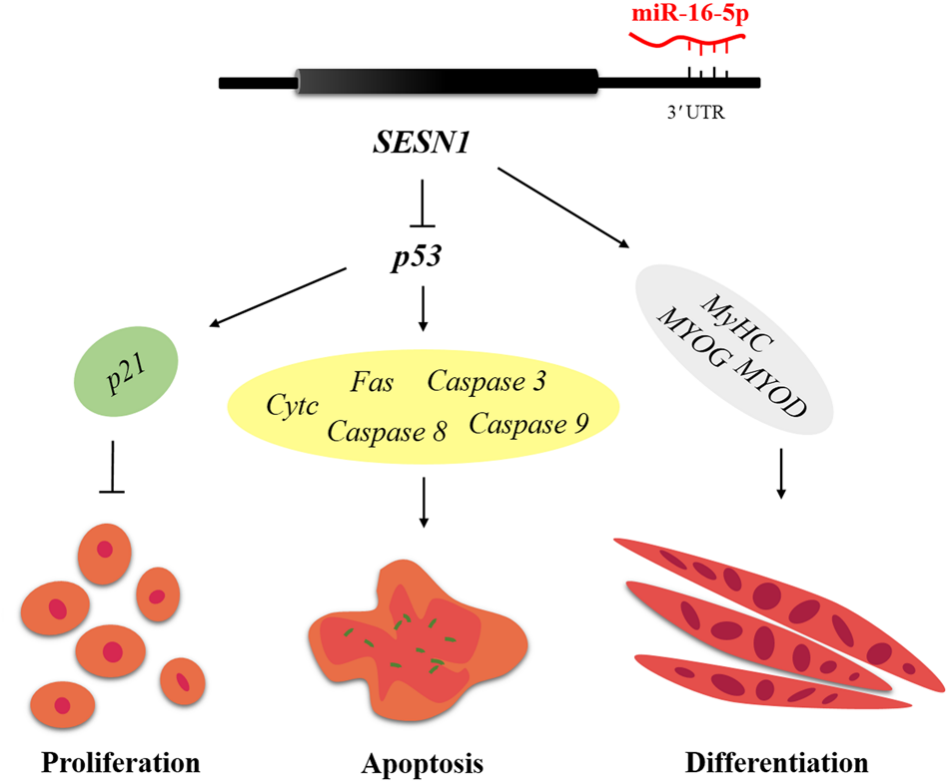
Model of miR-16-5p-mediated regulatory network for myoblast proliferation, apoptosis, and differentiation. Briefly, miR-16-5p inhibits expression of both mRNA and protein of *SESN1* by directly binding to the 3′ UTR of *SESN1*. *SESN1* can participate in the regulation of the *p53* signaling pathway by feedback control of *p53*, thus promoting and repressing myoblast proliferation and apoptosis, respectively. Meanwhile, *SESN1* is involved in myoblast differentiation

The present study reveals a role for miR-16-5p in myoblast proliferation, apoptosis, and differentiation. Our findings are partly based on the function of miR-16-5p in suppressing *SESN1* that regulates the proliferation and apoptosis of myoblast via the *p53* signaling pathway, as well as *p53*-independent myoblast differentiation (Fig. 8).

Dicer is an RNase III endonuclease, which is necessary for the processing of pre-miRNAs to mature miRNAs. In mice, Cre-mediated loss of Dicer resulted in reduced muscle miRNAs, decreased muscle fiber numbers, and myogenic cell apoptosis, which suggested that miRNAs play important role in the development of skeletal muscle^26^. There is evidence that miR-16-5p is involved in growth and development, as well as disease occurrence^27–30^. In our previous miRNA sequencing data, we found that miR-16-5p was differentially expressed between WRR and XH chickens. Here, we demonstrated a transient expression pattern of miR-16-5p during embryonic skeletal muscle development, and found that it was upregulated during chicken muscle fiber formation, indicating its potential role in muscle development. In order to investigate the biological functions of miR-16-5p in muscle development, we performed a series of in vitro experiments. We found that overexpression of miR-16-5p inhibited myoblasts proliferation, promoted myoblasts apoptosis, and repressed myoblast differentiation.

Because each miRNA could regulate up to hundred target genes^31^, we performed an in silico analysis of miRNA-16-5p targets, and identified that the seed sequence of miR-16-5p could perfectly match the 3′ UTR of *SESN1*, which was also differentially expressed between WRR and XH chickens. Moreover, the relative luciferase activity was significantly decreased with the overexpression of miR-16-5p. Furthermore, our rescue test also showed that co-overexpression of miR-16-5p and *SESN1* could neutralize the regulatory roles of miR-16-5p in myoblasts. In general, *SESN1* has been identified as a direct target of miR-16-5p, and miR-16-5p regulates myoblast development by suppressing *SESN1*.

*SESN1* is known to control the production of peroxiredoxins^32^. Recent research has shown that the repression of *SESN1* could play a critical role in Ras-induced ROS upregulation, and is involved in the regulation of cell activities, including proliferation, survival, and differentiation^33^. In another study, a gene ontology analysis of biological processes was performed using the Database for Annotation, Visualization and Integrated Discovery (DAVID) functional association database, and *SESN1* was found to be significantly enriched in functions related to the cell cycle and apoptosis^34^. Our results showed a constantly increased expression of *SESN1* during chicken skeletal muscle development, and was highly expressed in muscle tissue, which revealed that *SESN1* may play an important role in the process of muscle growth and development. *SESN1* overexpression significantly promoted proliferation of myoblast, and reduced cell apoptosis. In the meantime, overexpression of *SESN1* increased the expression of *MYOG*, *MYOD*, and *MyHC*, and promoted the differentiation of myoblast. Taken together, these results demonstrated that *SESN1* was involved in the regulation of myoblast proliferation, apoptosis, and differentiation that affected the growth and development of skeletal muscle.

*SESN1* was first identified in 1994 as a *p53* response gene^35^. *p53* is a sensor of genotoxic stress that could protect cells from DNA damage by inducing cell-cycle arrest^36^. It is well known that *p53*-mediated cell cycle arrest is caused mainly by *p53*-dependent transcription of *p21*, which inhibits cyclin-CDK complexes and phosphorylation of Rb tumor suppressor gene, thereby preventing cells from entering G1 phase^37^. As a tumor suppressor, *p53* also plays a crucial role in cell apoptosis^38^. Numerous studies have shown that *p53* induces apoptosis via both transcriptional activation of pro-apoptotic and repression of anti-apoptotic genes, as well as non-transcriptional mechanisms^39,40^. Interestingly, in our study, *SESN1*, which was previously described to be *p53*dependent^41^, was found to exert feedback regulation of *p53* by modulating the repair of DNA damage. Overexpression of *SESN1* inhibited the expression of both *p53* mRNA and protein, whereas loss of its function promoted *p53* upregulation. We also detected several downstream genes of the *p53* signaling pathway that were involved in cell proliferation (e.g., *p21*) and apoptosis (e.g., *Cyt c*, *Fas*, *Caspase 8*, *Caspase 3*, and *Caspase 9*), and found that the expressions of these downstream genes were differentially expressed with *SESN1* overexpression or knockdown. These observations strongly indicate that *SESN1* could regulate the *p53* signaling pathway by a feedback mechanism to promote and repress myoblast proliferation and apoptosis, respectively.

In conclusion, our findings reveal that miR-16-5p could inhibit myoblast proliferation, promote myoblast apoptosis, and repress myoblast differentiation by directly binding and suppressing *SESN1* expression. *SESN1* could regulate *p53* by a feedback mechanism, thereby participating in the regulation of *p53* signaling pathway, which suggested that this feedback control was important for myoblast proliferation and apoptosis. In addition, *SESN1* plays an active role in myoblast differentiation. Our findings present a novel model that elucidates the regulatory mechanism of how miR-16-5p controls muscle development. These findings provide a solid foundation for the understanding of the mechanisms and regulatory networks of myoblast proliferation, apoptosis, and differentiation, and will contribute to the development of further research.

## Materials and methods

### Ethics statement

All animal experimental protocols in this study were carried out in strict accordance with the regulations for the Administration of Laboratory Animals of Guangdong Province. The Institutional Animal Care and Use Committee at the South China Agricultural University approved the experiments (approval ID: SCAU#0011), and all efforts were made to minimize animal suffering.

### Experimental animals and tissues

Four Xinghua female chickens at 7 weeks of age were hatched from the Avian Farm of South China Agriculture University (Guangzhou, China). A total of 15 tissues, including cerebrum, cerebellum, hypothalamus, pituitary, heart, liver, spleen, lung, kidney, stomach muscle and glands, breast muscle, leg muscle, abdominal fat, and subcutaneous fat were collected as in our previous study^42^.

Three female chickens at each stage from E10 to E20, and 1 day (1D) were obtained from the same avian farm. Breast muscle tissues were used to detect the expression of miR-16-5p and *SESN1* in the process of chicken embryonic development.

### Cell culture

*DF-1 cells culture*: DF-1 cell line of chicken embryo fibroblast were cultured in DMEM (Gibco, USA) supplemented with 10% (v/v) fetal bovine serum (FBS, Hyclone, USA) and 0.2% penicillin/streptomycin (Invitrogen, USA).

*QM-7 cell culture*: QM-7 cell lines of avian myogenic origin were cultured in high-glucose M199 medium (Gibco, USA) with 10% fetal bovine serum (FBS), 10% tryptose phosphate broth solution (Sigma, USA), and 0.2% penicillin/streptomycin.

*CPM isolation and culture*: Primary myoblasts were isolated from the leg muscle of 11-day old chicken embryos. First, the muscle tissues were dissected away from the skin and bone, and then homogenized in a petri dish. To release single cells, the suspension was digested with pancreatin for 20 min at 37 °C. After neutralization with complete medium, single cells were collected by centrifugation at 500 × *g*. Subsequently, serial plating was performed to enrich for primary myoblasts and eliminate fibroblasts. Primary myoblasts were cultured in Roswell Park Memorial Institute (RPMI)-1640 medium (Gibco, USA) with 20% FBS, 1% nonessential amino acids, and 0.2% penicillin/streptomycin. The differentiation of myoblasts was induced by RPMI-1640 medium supplemented with 0.2% penicillin/streptomycin.

All cells were cultured at 37 °C in a 5% CO_2_ humidified atmosphere.

### RNA isolation, complementary DNA (cDNA) synthesis, and real-time (RT) PCR analysis

Total RNA was extracted from tissues or cells using Trizol reagent (TaKaRa, Japan) according to the manufacturer’s instructions. The integrity and concentration of all obtained RNA samples were assayed by 1.5% agarose gel electrophoresis, and determined by measuring the optical density in a Nanodrop 2000c spectrophotometer (Thermo, Waltham, MA, USA) at 260/280 nm ratio.

cDNA synthesis for mRNA was carried out using the PrimeScript RT Reagent Kit with gDNA Eraser (Perfect Real Time) (TaKaRa, Japan), which was able to eliminate genomic DNA. For miRNA, BμLge-Loop™ miRNA qRT-PCR Primer specific for gga-miR-16-5p and U6 were designed by RiboBio (RiboBio, Guangzhou, China), and ReverTra Ace qPCR RT Kit (Toyobo, Japan) was used to synthesize cDNA.

The RT-qPCR reactions were carried out in a Bio-Rad CFX96 Real-Time Detection instrument (Bio-Rad, Hercules, CA, USA). The iTaq Universal SYBR Green Supermix Kit (Toyobo, Japan) was used for cDNA quantification, according to the indicated manufacturer’s protocol. Chicken *β*-actin and U6 were used as internal controls. As described previously, data analysis was carried out with the comparative 2^−ΔΔCT^ method^43^.

### Primers

Primers were designed using Premier Primer 5.0 software (Premier Biosoft International, Palo Alto, CA, USA), and synthesized by Sangon Biotech (Shanghai, China). The major primers used in this study are listed in Table 1. Primers for RT-qPCR are shown in Table 2.

**Table 1:** Primers used for vector construction

Primer name	Primer sequences (5′ to 3′)	Size (bp)	Annealing temperature (◦C)
pcDNA3.1-*SESN1*	F: CTA**GCTAGC**ATGGAGGAGCGGGAGGGC	1713	60
	R: CCG**CTCGAG**TCAGGTCATATAGCGAGTAATA		
pmirGLO-*SESN1*-WT	F:CCG**CTCGAG**TAAGGCAAACGCAACAGG	357	55
	R:ACGC**GTCGAC**TCCAAATAACGCCGAACA		
pmirGLO-*SESN1*-MT	F:AAAATCTTGATATCAGTATTTGTGCTGGTG GAGCAAACAG	3028	56
	R:TTTTAGAACTATAGTCATAAACACGACCACCTCGTTTGTC		

Sequences in bold represent the enzyme cutting sites

**Table 2:** Primers used for real-time PCR

Gene name	Primer sequences (5′ to 3′)	Size (bp)	Annealing temperature (◦C)	Accession number
*SESN1*	F:CCGCTCCCTCTTCATTAC	123	55	XM_004940327.1
	R:CAACCATTTCGGGTCTCC			
*p53*	F:GAGATGCTGAAGGAGATCAATGAG	145	59	NM_205264.1
	R:GTGGTCAGTCCGAGCCTTTT			
*p21*	F:CCCGTAGACCACGAGCAGAT	102	61	NM_204396.1
	R:CGTCTCGGTCTCGAAGTTGA			
*Fas*	F:TCCACCTGCTCCTCGTCATT	78	61	NM_001199487.1
	R:GTGCAGTGTGTGTGGGAACT			
*Caspase-8*	F:CCCTGAAGACAGTGCCATTT	106	61	NM_204592.2
	R:GGGTCGGCTGGTCATTTTAT			
*CytC*	F:TGTCCAGAAATGTTCCCAGTGC	138	61	NM_001079478.1
	R:CCTTTGTTCTTATTGGCATCTGTG			
*Caspase-9*	F:TCCCGGGCTGTTTCAACTT	207	61	XM_424580.5
	R:CCTCATCTTGCAGCTTGTGC			
*Caspase-3*	F:TGGCCCTCTTGAACTGAAAG	139	61	NM_204725.1
	R:TCCACTGTCTGCTTCAATACC			
*MYOG*	F:CGGAGGCTGAAGAAGGTGAA	320	53	NM_204184.1
	R:CGGTCCTCTGCCTGGTCAT			
*MYOD*	F:GCTACTACACGGAATCACCAAAT	200	53	NM_204214.2
	R:CTGGGCTCCACTGTCACTCA			
*MyHC*	F:CTCCTCACGCTTTGGTAA	213	53	NM_001319304.1
	R:TGATAGTCGTATGGGTTGGT			
*β-actin*	F:GATATTGCTGCGCTCGTTG	194	50–65	NM_205518.1
	R:TTCAGGGTCAGGATACCTCTTT			

### RNA oligonucleotides and plasmids construction

Gga-miR-16-5p mimic, mimic NC, gga-miR-16-5p inhibitor, inhibitor NC, small interfering RNAs (siRNAs) used for the knockdown of *SESN1*, and non-specific siRNA negative control were designed and synthesized by RiboBio (Guangzhou, China). Oligonucleotide sequences in this study are showed in Table 3.

**Table 3:** Oligonucleotide sequences in this study

Fragment name	Sequences (5′ to 3′)
miR-16-5p mimic	UAGCAGCACGUAAAUAUUGGUG
miR-16-5p inhibitor	CACCAAUAUUUACGUGCUGCUA
si-*SESN1*	CCTTGCTTCCTTCACGTTT

For *SESN1* overexpression plasmid construction, the full-length sequence of *SESN1* was amplified from chicken breast muscle cDNA by PCR, and cloned into the expression plasmid, pcDNA-3.1 vector (Promega, Madison, WI, USA) by using the *NheI* and *XhoI* restriction sites. For the pmirGLO dual-luciferase miRNA target reporter vector, the segment sequence of the *SESN1* 3′ UTR that contained the putative gga-miR-16b-5p binding sequence was amplified by PCR, and then subcloned into *XhoI* and *SalI* restriction sites in the pmirGLO dual-luciferase reporter vector (Promega, Wisconsin, USA). The *SESN1*-3′ UTR mutant plasmid was generated by changing the binding site of miR-16-5p from TGCTGCT to TATCAGT. PCR amplification was performed for the mutagenesis and *DpnI* digestion to remove the parental DNA.

### Cell transfection

All transient transfections used Lipofectamine 3000 Reagent (Invitrogen, USA) following the manufacturer’s protocol. For RNA oligonucleotides, a concentration of 100 nM was used.

### Dual-luciferase reporter assay

DF-1 cells were seeded in a 96-well plate. After being co-transfected with pmirGLO-WT-*SESN1*-3′ UTR (wild type) or pmirGLO-MT-*SESN1*-3′ UTR (mutant type) plasmids, or gga-miR-16-5p mimic and mimic NC, firefly and *Renilla* luciferase activities were measured at 48 h post transfection using a Dual-GLO Luciferase Assay System Kit (Promega, USA), following the manufacturer’s instructions. Luminescence was measured using a Fluorescence/Multi-Detection Microplate Reader (BioTek, USA) and firefly luciferase activities were normalized to Renilla luminescence in each well.

### Western blotting assay

Cellular protein was extracted using radio immune precipitation assay (RIPA) buffer (Beyotime, China) with phenylmethane sulfonyl fluoride (PMSF) protease inhibitor (Beyotime, China). After incubation on ice for 10 min, the supernatant was collected by centrifugation at 10,000 × *g* for 5 min at 4◦C. The proteins were separated in 12% SDS-PAGE and transferred onto nitrocellulose membranes (Whatman, Maidstone, UK), and then probed with antibodies following standard procedures. The antibodies and their dilutions used for western blots were as follows: Anti-PA26/SESN1 antibody (aa375-424) LS-C145280 (LS-C145280/76757; LifeSpan Biosciences, USA; 1 μg/ml), purified mouse anti-p53 (554166; BD Biosciences, USA; l:1,000), myogenin antibody (orb6492; Biorbyt, UK; 1:100), B103 (DHSB, USA; 0.5 μg/ml), p21 Cip1 antibody (GTX112898; GeneTex, USA; 1:500), Anti-gamma H2A.X (phospho S139) antibody [9F3] (ab26350; Abcam, UK; 1 μg/ml), Actived-Caspase-3 p17 polyclonal antibody (BS7004; Bioworld, USA; 1: 500) Cleaved Caspase-8 (Asp391) (18C8) Rabbit mAb (9496; Cell Signaling Technology, USA; 1:1000), Anti-Caspase-9 antibody [E23] (ab32539; Abcam, UK; 1:1000), rabbit anti-GAPDH (AB-P-R 001; Hangzhou Goodhere Biotechnology Co. Ltd., China; 1:1,500), goat anti-rabbit IgG-HRP (BA1054; Boster, China; 1:5,000) and peroxidase-goat anti-mouse IgG (BA1051; Boster, China; 1:2,500).

### EdU assay

Cells seeded in 24-well plates were cultured to 50% density and then transfected. Forty-eight hours after transfection with mimics, inhibitors, overexpression vector, or siRNAs, the cells were fixed and stained with a C10310 EdU Apollo In Vitro Imaging Kit (RiboBio, China) as previously described^42^. A fluorescence microscope (TE2000-U; Nikon, Japan) was used to capture three randomly selected fields to visualize the number of EdU-stained cells.

### Flow cytometric analysis of the cell cycle

The cultured cells in growth media were collected after a 48-h transfection and then fixed in 70% ethanol overnight at −20 °C. Subsequently, the fixed cells were stained with propidium iodide (Sigma, USA) (50 μg/ml) containing 10 μg/ml RNase A (Takara, USA) and 0.2% (v/v) Triton X-100 (Sigma), and then incubated for 30 min at 37  °C in the dark. Flow cytometric analysis was performed on a BD AccuriC6 flow cytometer (BD Biosciences, USA) and data was processed using FlowJo7.6 software.

### CCK-8 assay

Cells were seeded in a 96-well plate and cultured in growth medium. After being transfected, cell proliferation was monitored at 12 h, 24 h, 36 h, and 48 h using a TransDetect CCK (TransGen Biotech, Beijing, China), according to the manufacturer’s protocol. Absorbance at 450 nm was measured using a Model 680 Microplate Reader (Bio-Rad) after 1 h of incubation.

### Cell apoptosis assay

At 48 h after transfection, the percentage of apoptotic cells was measured using an AnnexinV FITC apoptosis detection kit (BD Biosciences, USA) and analyzed by flow cytometry (BD Biosciences, USA), according to the indicated manufacturer’s protocol.

### Immunofluorescence

For immunofluorescence, cells were seeded in 24-well plates. After transfection for 48 h, cells were fixed in 4% formaldehyde for 20 min then washed three times with PBS for 5 min. Subsequently, the cells were permeabilized by adding 0.1% Triton X-100 for 15 min and blocked with goat serum for 30 min. After incubation with *MyHC* (B103; DHSB, USA; 0.5 μg/ml) overnight at 4 °C, the Fluorescein (FITC)-conjugated AffiniPure Goat Anti-Mouse IgG (H + L) (BS50950; Bioworld, USA; 1:50) was added and the cells were incubated at room temperature for 1 h. The cell nuclei were stained with DAPI for 5 min. Images were obtained with a fluorescence microscope (TE2000-U; Nikon, Japan). The area of cells labeled with anti-*MyHC* was measured by using ImageJ software (National Institutes of Health), and the total myotube area was calculated as a percentage of the total image area covered by myotubes.

### Statistical analysis

All data are presented as mean ± standard error of the mean (S.E.M.) based on at least three independent experiments for each treatment. Unpaired Student’s *t*-test was used for *P*-value calculations, and *P* < 0.05 was considered significant.

## Electronic supplementary material

Supplementary File 1. Differential expression analysis in breast muscle between WRR and XH chicken by RNA sequencing
