# Supplementary material for: MiR-16-5p targets SESN1 to regulate the p53 signaling pathway, affecting myoblast proliferation and apoptosis, and is involved in myoblast differentiation
Source: Cell Death Dis. 2018 Mar 6;9(3):367. doi: 10.1038/s41419-018-0403-6 (PMC5840423; doi:10.1038/s41419-018-0403-6)
Supplement: Supplementary file 1 — Differential expression analysis in breast muscle between WRR and XH chicken by RNA sequencing [file 41419_2018_403_MOESM1_ESM.docx]

**Supplementary File 1. Differential expression analysis in breast muscle between WRR and XH chicken by RNA sequencing.**

| **miRNA/Gene** | **WRR-FPKM** | **XH-FPKM** | **log2(WRR/XH)** | **q value** | **Regulation^a^** |
| --- | --- | --- | --- | --- | --- |
| gga-miR-16-5p | 56158.77 | 85642.55 | -0.599249972 | 0 | Down |
| *SESN1* | 622.095 | 228.65 | 1.443993992 | 0.00016231 | up |

a. "Up" means miRNA was up-regulated in WRR chicken compared to XH chicken. "Down" means miRNA was down-regulated in WRR chicken compared to XH chicken.
